# Dementia Knowledge and Care Experience of Physicians in The South‐South Region of Nigeria: A Mixed Method Study

**DOI:** 10.1002/alz70858_102824

**Published:** 2025-12-25

**Authors:** Carter O Okolakpa, Gideon N Inyangudo, Lorelei Jones

**Affiliations:** ^1^ Delta State University Teaching Hospital, Oghara, Delta, Nigeria; ^2^ Institute of Human Virology, Nigeria, Lagos, Lagos, Nigeria; ^3^ Bangor University, Wales, Wales, United Kingdom

## Abstract

**Background:**

Physicians play a significant role in the diagnosis and treatment for people with dementia ^[1]^. However, little is known about physicians’ capacity and experience in dementia care, especially in low‐ and middle‐income countries like Nigeria. The aim of the present study was to assess physicians’ dementia knowledge and diagnostic approach in the south‐south region of Nigeria.

**Method:**

This pilot study was a cross‐sectional mixed study conducted among fifteen (15) physicians across five healthcare facilities in the south‐south region of Nigeria. Quantitative data comprising of socio‐demographics and dementia knowledge scales were collected using a self‐administered questionnaire. Key informant interviews (KIIs) exploring the experience of physicians in the diagnosis of dementia were also conducted among participants.

**Result:**

Fifteen physicians were purposively recruited for this study. The overall mean knowledge score of participants was 17.67 ± 3.29 out of a maximum possible score of 25. About half (53.3%) of study respondents had good dementia knowledge assessment scores. Care experienced by physicians from the qualitative findings was grouped into five themes to include: 1) Rate of dementia cases seen at the facility with low cases of dementia patients reported 2) Tools used for diagnosis of dementia with the Mini Mental State Examination (MMSE) and Montreal Cognitive Assessment are commonly reported. 3) Training of physicians on assessment and management of dementia cases with no formal training reported by participants 4) Clinical investigations conducted for dementia patients with MRI and CT were commonly reported 5) Communication of the diagnosis of dementia with the patients and their caregivers

**Conclusion:**

Routine update training to enhance the capacity of healthcare professionals in the diagnosis and management of dementia cases is needed. Also, healthcare facilities should be equipped with cutting‐edge facilities to enhance diagnosis and management of dementia.

